# Intracellular Proteolysis of Progranulin Generates Stable, Lysosomal Granulins that Are Haploinsufficient in Patients with Frontotemporal Dementia Caused by *GRN* Mutations

**DOI:** 10.1523/ENEURO.0100-17.2017

**Published:** 2017-08-18

**Authors:** Christopher J. Holler, Georgia Taylor, Qiudong Deng, Thomas Kukar

**Affiliations:** 1 Department of Pharmacology, Emory University School of Medicine, Atlanta, GA 30322; 2 Department of Biochemistry, Emory University School of Medicine, Atlanta, GA 30322; 3 Center for Neurodegenerative Disease, Emory University School of Medicine, Atlanta, GA 30322; 4 Department of Neurology, Emory University School of Medicine, Atlanta, GA 30322

**Keywords:** Alzheimer's disease, amyotophic lateral sclerosis, autophagy, cathepsin L, frontotemporal dementia, granulins, lysosomal storage disease, neurodegeneration, neuroinflammation, neuronal ceroid lipofuscinosis, Parkinson's disease, progranulin

## Abstract

Homozygous or heterozygous mutations in the *GRN* gene, encoding progranulin (PGRN), cause neuronal ceroid lipofuscinosis (NCL) or frontotemporal dementia (FTD), respectively. NCL and FTD are characterized by lysosome dysfunction and neurodegeneration, indicating PGRN is important for lysosome homeostasis in the brain. PGRN is trafficked to the lysosome where its functional role is unknown. PGRN can be cleaved into seven 6-kDa proteins called granulins (GRNs); however, little is known about how GRNs are produced or if levels of GRNs are altered in FTD-*GRN* mutation carriers. Here, we report the identification and characterization of antibodies that reliably detect several human GRNs by immunoblot and immunocytochemistry. Using these tools, we find that endogenous GRNs are present within multiple cell lines and are constitutively produced. Further, extracellular PGRN is endocytosed and rapidly processed into stable GRNs within lysosomes. Processing of PGRN into GRNs is conserved between humans and mice and is modulated by sortilin expression and mediated by cysteine proteases (i.e. cathpesin L). Induced lysosome dysfunction caused by alkalizing agents or increased expression of transmembrane protein 106B (TMEM106B) inhibit processing of PGRN into GRNs. Finally, we find that multiple GRNs are haploinsufficient in primary fibroblasts and cortical brain tissue from FTD-*GRN* patients. Taken together, our findings raise the interesting possibility that GRNs carry out critical lysosomal functions and that loss of GRNs should be explored as an initiating factor in lysosomal dysfunction and neurodegeneration caused by *GRN* mutations.

## Visual Abstract

**Figure:**
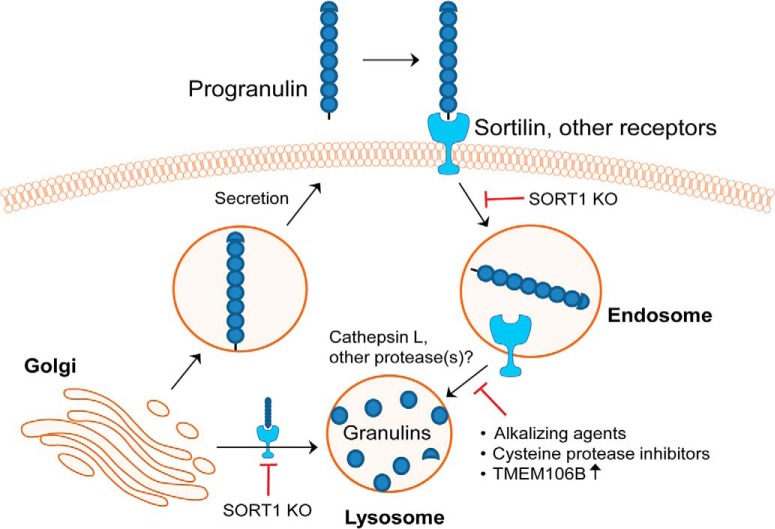

## Significance Statement

Progranulin (PGRN) plays a critical, yet undefined role in lysosome function. PGRN is cleaved into 6-kDa proteins called granulins (GRNs), but this process is poorly understood. We find that PGRN is proteolytically processed into stable, lysosomal GRNs, implying that GRNs may have a functional role in the lysosome, and are not toxic as previously proposed. Moreover, deficiency of GRNs in frontotemporal dementia (FTD) caused by *GRN* mutations may play a causal role in the development of lysosome dysfunction that underlies FTD-*GRN*, which paves the way for testing GRN replacement as a therapeutic strategy. Finally, potential drug candidates to treat FTD-*GRN* should evaluate their effect on the production of both PGRN and GRNs in the brain.

## Introduction

Progranulin (PGRN) is a ∼88-kDa multifunctional, secreted glycoprotein that is ubiquitously expressed. PGRN has an important function in the brain, where it is expressed primarily in microglia and neurons (Suh et al., 2012; Uhlén et al., 2015; Zhang et al., 2016). Notably, PGRN is composed of seven ∼6-kDa granulin (GRN) proteins and one half-granulin protein termed paragranulin (para-GRN). Each GRN protein shares an evolutionary conserved cysteine-rich consensus motif and is folded into a similar structure stabilized by multiple disulfide bonds (Hrabal et al., 1996; Tolkatchev et al., 2008). Within PGRN, each GRN is joined by short linear sequences, termed linkers, which can be cleaved by proteolysis to release the mature GRN proteins (Zhu et al., 2002; Kessenbrock et al., 2008; Suh et al., 2012). The GRNs were originally named using letters (A-G plus para-GRN) when they were first discovered (Bateman et al., 1990). The consensus nomenclature (UniProtKB: P28799) refers to each GRN numerically according to their position within PGRN starting at the amino (N) terminus as follows: para-GRN, GRN-1 (G), GRN-2 (F), GRN-3 (B), GRN-4 (A), GRN-5 (C), GRN-6 (D), GRN-7 (E). The functional roles of GRNs are unknown, in part, because specific antibodies to detect endogenous GRNs have not been available.

PGRN and GRNs came to the attention of the neuroscience field in 2006, when autosomal dominant mutations in the *GRN* gene were discovered as a common cause of frontotemporal dementia (FTD) with inclusions of the TAR DNA-binding protein 43 (TDP-43; Baker et al., 2006; Cruts et al., 2006; Gass et al., 2006). FTD is the most common type of dementia in people under 60 years of age and is the clinical term for a spectrum of incurable neurodegenerative diseases affecting the frontal and temporal lobes (Bang et al., 2015). *GRN* mutations cause FTD through haploinsufficiency or loss-of-function of PGRN (Ghidoni et al., 2012a; Kleinberger et al., 2013; Pottier et al., 2016). In FTD-*GRN* carriers, circulating PGRN levels are decreased by ∼50% in plasma and CSF (Finch et al., 2009; Ghidoni et al., 2012b; Meeter et al., 2016). However, it is unknown how *GRN* mutations affect levels of GRNs in the brain. Moreover, it is unclear why loss of PGRN in the brain causes neurodegeneration.

One potential explanation, with increasing support, is that PGRN haploinsufficiency causes lysosome dysfunction (Sargeant, 2016). Lysosome dysfunction is a common occurrence in numerous neurodegenerative diseases (Platt et al., 2012; Martini-Stoica et al., 2016) and growing evidence indicates a critical role for PGRN in the maintenance of lysosome homeostasis (Ahmed et al., 2010; Smith et al., 2012; Götzl et al., 2014; Tanaka et al., 2014; Almeida et al., 2016; Lui et al., 2016). First, PGRN is associated with the lysosome based on proteomic, transcriptomic, and immunofluorescence studies (Kollmann et al., 2005; Hu et al., 2010; Settembre et al., 2011; Song et al., 2013; Gowrishankar et al., 2015). Further, a portion of PGRN can be trafficked to the lysosome through either a sortilin (SORT1; Hu et al., 2010)- or prosaposin (PSAP; Zhou et al., 2015)-dependent pathway. Additionally, humans with homozygous *GRN* mutations that make no PGRN develop a lysosomal storage disease called neuronal ceroid lipofuscinosis (NCL; Smith et al., 2012; Canafoglia et al., 2014; Almeida et al., 2016). Homozygous *Grn* knockout (KO) mice have defects similar to NCL including neuroinflammation, lipofuscin accumulation, and lysosome dysfunction (Ahmed et al., 2010; Wils et al., 2012; Götzl et al., 2014; Sargeant, 2016). Importantly, NCL-like lysosomal dysfunction and pathology has been observed in cells and brain tissue from heterozygous FTD-*GRN* patients (Götzl et al., 2014; Ward et al., 2017), suggesting a common underlying disease process. Finally, the transmembrane protein 106B (TMEM106B), a neuronal lysosomal protein that regulates lysosome transport and function, is a strong genetic risk factor for FTD-*GRN* (Van Deerlin et al., 2010; Finch et al., 2011; Nicholson and Rademakers, 2016). Thus, determining how PGRN and GRNs affect lysosome function is critical to understanding the pathogenesis of diseases caused by *GRN* mutations.

GRNs were identified from extracts of cells and various tissues using large-scale biochemical fractionation over twenty years ago, yet are still poorly understood (Bateman et al., 1990; Shoyab et al., 1990; Belcourt et al., 1993). Previously, GRNs were proposed to be produced from extracellular PGRN, especially in inflammatory conditions (Zhu et al., 2002; Kessenbrock et al., 2008), although this has not been demonstrated *in vivo*. Alternatively, based on the emerging lysosomal role of PGRN, we hypothesized that GRNs are produced intracellular in the lysosome. Unfortunately, the lack of sensitive tools to detect GRNs has hampered the ability to test this idea. Here, we report for the first time, the identification and characterization of antibodies that reliably detect several human GRNs by immunoblot and immunocytochemistry. Using these tools, we have begun to dissect the intracellular production and localization of GRNs. Further, we provide evidence that multiple GRNs, like their precursor PGRN, are haploinsufficient in FTD-*GRN* mutation patients.

## Materials and Methods

### Statistical analysis

Details of the statistical analysis used for each experiment are listed in Table 1. All data are presented as the mean ± SEM. An unpaired two-tailed Student’s *t* test was used to generate *p* values for experiments where two groups were compared. One-way ANOVA was used followed by Dunnett’s comparison *post hoc* test for comparisons of more than two groups. Statistical analyses were performed in GraphPad Prism 6.02 (GraphPad Software); *p* < 0.05 was considered significant.

**Table 1.** Statistical analysis

**Figure**	**Structure of the data**	**Type of test**	***p* value**	**95% confidence intervals**
Fig. 6C, intracellular PGRN in WT vs SORT1-KO lysates (*n* = 3 independent replicates)	Normal distribution	Unpaired *t* test	*p* = 0.0196	30.13 to 198.5
Fig. 6D, intracellular GRN-2,3 in WT vs SORT1-KO lysates (*n* = 3 independent replicates)	Normal distribution	Unpaired *t* test	*p* = 0.0486	-93.93 to -0.4729
Fig. 6*G*, intracellular PGRN in TMEM106B-expressing HeLa lysates (*n* = 3 independent replicates)	Normal distribution	Unpaired *t* test	*p* = 0.0005	91.99 to 157.9
Fig. 6*H*, intracellular GRN-2,3 in TMEM106B-expressing HeLa lysates (*n* = 3 independent replicates)	Normal distribution	Unpaired *t* test	*p* = 0.0426	-134.8 to -3.742
Fig. 6I, secreted PGRN in TMEM106B-expressing HeLa cell media (*n* = 3 independent replicates)	Normal distribution	Unpaired *t* test	*p* = 0.4803	-25.64 to 45.58
Fig. 6*L*, intracellular PGRN in TMEM106B-expressing PGRN KO cells pulsed with PGRN (*n* = 3 independent replicates)	Normal distribution	Unpaired *t* test	*p* = 0.0055	36.84 to 113.6
Fig. 6*M*, intracellular GRN-2,3 in TMEM106B-expressing PGRN KO cells pulsed with PGRN (*n* = 3 independent replicates)	Normal distribution	Unpaired *t* test	*p* = 0.0282	-107.5 to -10.32
Fig. 7A, secreted PGRN in treated HAP1 cell media (*n* = 3 independent replicates)	Normal distribution	One-way ANOVA	*p* = 0.0009, *p* < 0.0001, *p* < 0.0001	-602.5 to -207.5; -1774 to -1380; -1865 to -1471
Fig. 7*C*, intracellular PGRN in treated HAP1 cell lysates (*n* = 3 independent replicates)	Normal distribution	One-way ANOVA	*p* = 0.0007, *p* = 0.0332, *p* = 0.0151	-417.2 to -153.8; -276.1 to -12.74; -301.2 to -37.84
Fig. 7*D*, intracellular GRN-2,3 in treated HAP1 cell lysates (*n* = 3 independent replicates)	Normal distribution	One-way ANOVA	*p* < 0.0001 for all 3 treatments	62.35 to 104.7; 65.25 to 107.6; 56.25 to 98.55
Fig. 7*I*, intracellular PGRN in cathepsin L inhibitor-treated HAP1 cell lysates (*n* = 3 independent replicates)	Normal distribution	One-way ANOVA	*p* > 0.9999, **p**= 0.5501, *p* = 0.1055	-39.01 to 37.81; -54.91 to 21.91; -70.11 to 6.712
Fig. 7*J*, intracellular GRN-2,3 in cathepsin L inhibitor-treated HAP1 cell lysates (*n* = 3 independent replicates)	Normal distribution	One-way ANOVA	*p* = 0.2116, *p* = 0.0144, *p* = 0.0003	62.35 to 104.7; 65.25 to 107.6; 56.25 to 98.55
Fig. 9B, intracellular PGRN in human fibroblasts (3 independent patient lines for control and FTD-*GRN*)	Normal distribution	Unpaired *t* test	*p* = 0.0030	-71.47 to -28.33
Fig. 9C, intracellular GRN-2,3 in human fibroblasts (3 independent patient lines for control and FTD-*GRN*)	Normal distribution	Unpaired *t* test	*p* = 0.0023	-75.34 to -31.98
Fig. 9*D*, intracellular GRN-4 in human fibroblasts (3 independent patient lines for control and FTD-*GRN*)	Normal distribution	Unpaired *t* test	*p* = 0.0176	-90.57 to -15.19
Fig. 9F, GRN-2,3 expression in human brain tissue (5 independent patient samples for control and FTD-*GRN*)	Normal distribution	Unpaired *t* test	*p* = 0.0123	-106.3 to -17.49
Fig. 9*G*, GRN-4 expression in human brain tissue (5 independent patient samples for control and FTD-*GRN*)	Normal distribution	Unpaired *t* test	*p* = 0.0005	-83.70 to -35.11
Fig. 9*H*, PGRN expression by ELISA in human brain tissue (5 independent patient samples for control and FTD-*GRN*)	Normal distribution	Unpaired *t* test	*p* = 0.0403	-78.11 to -2.261

### Chemical reagents

Bafilomycin A1 (BafA1) was from Tocris (R&D Systems). The inhibitors Z-FA-fmk (cysteine protease) and Z-VAD-fmk (pan-caspase) were from Calbiochem (EMD Millipore) and BD PharMingen (BD Biosciences), respectively. Cathepsin L inhibitor II (Z-FY-CHO) and ALLN were from Calbiochem. Elastase inhibitor MeOSuc-AAPV-cmk (N-(Methoxysuccinyl)-Ala-Ala-Pro-Val-chloromethyl ketone) and all other chemical inhibitors were from Sigma-Aldrich.

### Cloning of human GRN expression vectors

The DNA sequences for individual GRNs were codon optimized and custom synthesized by GenScript. First, the amino acid sequence for human para-GRN and each GRN (1 through 7), including the linker region at the carboxyl (C)-terminal end was identified based on the Universal Protein Resource database (P28799l; GRN_HUMAN; Table 2). The endogenous PGRN signal peptide (SP) sequence followed by a twin-Strep and FLAG tag was added to the N terminus of each GRN. Synthetic GRN gene constructs were designed to add a 5’ HindIII (AAGCTT) site, a Kozak sequence (GCCACC) before the ATG start codon, a 3’ Stop codon, and a XhoI (TGACTCGAG) site. Following synthesis, each gene was inserted into the pcDNA3.1 (+) vector using a HindIII/XhoI cloning strategy. All constructs were verified using DNA sequencing, restriction digests, and PCR amplification. Subsequently, primers were designed for each GRN to remove the linker region; each construct was amplified via PCR, and subcloned into pcDNA3.1 (+) vector, and verified using DNA sequencing.

**Table 2.** Human GRN expression constructs

**Construct**	**Amino acid sequence**
Para-GRN + linker-1	MWTLVSWVALTAGLVAG*SAWSHPQFEKGGGSGGGSGGSAWSHPQFEKGASDYKDDDDK*TRCPDGQFCPVACCLDPGGASYSCCRPLLDKWPTTLSRHL
Granulin-1 + linker 2	MWTLVSWVALTAGLVAG*SAWSHPQFEKGGGSGGGSGGSAWSHPQFEKGASDYKDDDDK*GGPCQVDAHCSAGHSCIFTVSGTSSCCPFPEAVACGDGHHCCPRGFHCSADGRSCFQRSGNNSVG
Granulin-2 + linker 3	MWTLVSWVALTAGLVAG*SAWSHPQFEKGGGSGGGSGGSAWSHPQFEKGASDYKDDDDK*AIQCPDSQFECPDFSTCCVMVDGSWGCCPMPQASCCEDRVHCCPHGAFCDLVHTRCITPTGTHPLAKKLPAQRTNRAVALSS
Granulin-3 + linker 4	MWTLVSWVALTAGLVAG*SAWSHPQFEKGGGSGGGSGGSAWSHPQFEKGASDYKDDDDK*SVMCPDARSRCPDGSTCCELPSGKYGCCPMPNATCCSDHLHCCPQDTVCDLIQSKCLSKENATTDLLTKLPAHTVG
Granulin-4 + linker 5	MWTLVSWVALTAGLVAG*SAWSHPQFEKGGGSGGGSGGSAWSHPQFEKGASDYKDDDDK*DVKCDMEVSCPDGYTCCRLQSGAWGCCPFTQAVCCEDHIHCCPAGFTCDTQKGTCEQGPHQVPWMEKAPAHLSLPDPQALKR
Granulin-5 + linker 6	MWTLVSWVALTAGLVAG*SAWSHPQFEKGGGSGGGSGGSAWSHPQFEKGASDYKDDDDK*DVPCDNVSSCPSSDTCCQLTSGEWGCCPIPEAVCCSDHQHCCPQGYTCVAEGQCQRGSEIVAGLEKMPARRASLSHPR
Granulin-6 + linker 7	MWTLVSWVALTAGLVAG*SAWSHPQFEKGGGSGGGSGGSAWSHPQFEKGASDYKDDDDK*DIGCDQHTSCPVGQTCCPSLGGSWACCQLPHAVCCEDRQHCCPAGYTCNVKARSCEKEVVSAQPATFLARSPHVGVK
Granulin-7 + linker 8	MWTLVSWVALTAGLVAG*SAWSHPQFEKGGGSGGGSGGSAWSHPQFEKGASDYKDDDDK*DVECGEGHFCHDNQTCCRDNRQGWACCPYRQGVCCADRRHCCPAGFRCAARGTKCLRREAPRWDAPLRDPALRQLL
Para-GRN – linker-1	MWTLVSWVALTAGLVAG*SAWSHPQFEKGGGSGGGSGGSAWSHPQFEKGASDYKDDDDK*TRCPDGQFCPVACCLDPGGASYSCCRPLLD
Granulin-1 – linker 2	MWTLVSWVALTAGLVAG*SAWSHPQFEKGGGSGGGSGGSAWSHPQFEKGASDYKDDDDK*GGPCQVDAHCSAGHSCIFTVSGTSSCCPFPEAVACGDGHHCCPRGFHCSADGRSCFQ
Granulin-2 – linker 3	MWTLVSWVALTAGLVAG*SAWSHPQFEKGGGSGGGSGGSAWSHPQFEKGASDYKDDDDK*AIQCPDSQFECPDFSTCCVMVDGSWGCCPMPQASCCEDRVHCCPHGAFCDLVHTRCIT
Granulin-3 – linker 4	MWTLVSWVALTAGLVAG*SAWSHPQFEKGGGSGGGSGGSAWSHPQFEKGASDYKDDDDK*SVMCPDARSRCPDGSTCCELPSGKYGCCPMPNATCCSDHLHCCPQDTVCDLIQSKCLS
Granulin-4 – linker 5	MWTLVSWVALTAGLVAG*SAWSHPQFEKGGGSGGGSGGSAWSHPQFEKGASDYKDDDDK*DVKCDMEVSCPDGYTCCRLQSGAWGCCPFTQAVCCEDHIHCCPAGFTCDTQKGTCEQ
Granulin-5 – linker 6	MWTLVSWVALTAGLVAG*SAWSHPQFEKGGGSGGGSGGSAWSHPQFEKGASDYKDDDDK*DVPCDNVSSCPSSDTCCQLTSGEWGCCPIPEAVCCSDHQHCCPQGYTCVAEGQCQR
Granulin-6 – linker 7	MWTLVSWVALTAGLVAG*SAWSHPQFEKGGGSGGGSGGSAWSHPQFEKGASDYKDDDDK*DIGCDQHTSCPVGQTCCPSLGGSWACCQLPHAVCCEDRQHCCPAGYTCNVKARSCEK
Granulin-7 – linker 8	MWTLVSWVALTAGLVAG*SAWSHPQFEKGGGSGGGSGGSAWSHPQFEKGASDYKDDDDK*DVECGEGHFCHDNQTCCRDNRQGWACCPYRQGVCCADRRHCCPAGFRCAARGTKCLR

Each construct contains the human PGRN signal peptide followed by a twin-strep/FLAG tag (italicized) before the GRN ± linker domains.

### Purification of recombinant PGRNs

A tandem affinity purification (TAP) tag was cloned onto the C terminus or N terminus of full-length human PGRN to generate C-TAP PGRN or N-TAP PGRN. C-TAP PGRN contains a twin-Strep tag followed by the FLAG epitope. N-TAP PGRN contains a twin Strep tag followed by a V5 epitope tag inserted following the endogenous PGRN signal peptide sequence. Stable HEK 293T cell lines overexpressing either C-TAP PGRN or N-TAP PGRN were generated. Stable cells were cultured and maintained in DMEM that contained 100 µg/ml Zeocin (Life Technologies/Thermo Fisher) and conditioned media were collected. PGRN was affinity purified from conditioned media over Strep-Tactin XT Superflow (catalog 2-4010-025) resin using a slightly modified protocol as described by the manufacturer (IBA GmbH). The mCherry-PGRN construct has been described (Hu et al., 2010). HEK Expi293 cells (RRID:CVCL_D615) were transfected with the mCherry-PGRN construct and conditioned media were collected following the manufacturer’s protocol (Thermo Fisher; catalog A14635). mCherry-PGRN contains a poly-histidine tag and was purified from the media over a cOmplete His-Tag column following manufacturer’s protocol (Sigma-Aldrich; catalog 05893682001). For all purifications, the elutions containing recombinant PGRNs were concentrated and desalted into PBS using Vivaspin 500 Protein Concentrators [molecular weight (MW) cutoff 50 kDa; catalog 28932218; GE Healthcare Life Sciences]. The purity of recombinant PGRN was assessed by SDS-PAGE followed by colloidal coomassie dye G-250 protein stain (GelCode Blue; Thermo Fisher) and estimated to be >95% pure.

### Cell culture

HeLa (human cervix carcinoma; American Type Culture Collection; ATCC), HEK293T (human embryonic kidney; ATCC; catalog CRL-3216, RRID:CVCL_0063 RRID), H4 (human neuroglioma; ATCC; RRID:CVCL_1239), and HEK293T cells stably expressing N-TAP PGRN (HEK-PGRN) were cultured in high glucose DMEM supplemented with 10% FBS, 1% penicillin/streptomycin, and 1% GlutaMax (Life Technologies). SH-SY5Y (human neuroblastoma; ATCC; catalog CRL-2266, RRID:CVCL_0019) and SW-13 (human adrenal cortical adenocarcinoma; ATCC) cells were cultured in DMEM/Ham’s F12 1:1 medium supplemented with 10% FBS and 1% penicillin/streptomycin. The near-haploid human cell line HAP1 (RRID:CVCL_Y019) has been previously described (Essletzbichler et al., 2014). The *GRN* KO (PGRN KO) HAP1 cell line (HZGHC004031c006) was produced using CRISPR/Cas9 gene editing to introduce a frame shift mutation into the coding sequence (10bp deletion in exon 2) of *GRN* (Horizon Discovery). The *SORT1* KO (SORT1 KO) HAP1 cell line (HZGHC001782c010) was produced using CRISPR/Cas9 gene editing to introduce a frame shift mutation into the coding sequence (1bp insertion in exon 5) of *SORT1* (Horizon Discovery). HAP1 cells were cultured in Iscove’s modified Dulbecco’s medium supplemented with 10% FBS and 1% penicillin/streptomycin. The *Grn* KO (PGRN KO) mouse embryonic fibroblast (MEF) cells were generated and kindly provided by Dr. Laura Reinholdt at The Jackson Laboratory and cultured in DMEM supplemented with 10% FBS, 1% penicillin/streptomycin, and 1% GlutaMax. Primary human fibroblasts from control and FTD-*GRN* patients were collected by skin punch biopsy and cultured under standard published protocols.

### Transfections

HEK293T cells or HAP1 PGRN KO cells were cultured as above in six-well dishes and transfected with 2 µg of plasmid DNA (empty vector or GRN constructs ± linkers) using Mirus LT1 transfection reagent (Mirus Bio, LLC) according to the manufacturer’s protocol. Cells were harvested for biochemical analysis or fixed and immunostained after 24 or 48 h. The TMEM106B constructs (AAV1 backbone; untagged or with C-terminal V5 tag) have been previously described (Nicholson et al., 2013). HeLa or HAP1 PGRN KO cells were plated in six-well plates and transfected with 2 µg of empty vector or TMEM106B for a total of 48 h. For the PGRN KO cells, 24 h after transfection, mCherry-PGRN (5 µg/ml) was added to the media for an additional 24 h before lysing the cells for SDS-PAGE and immunoblot analysis.

### Cell lysis and immunoblotting

For whole cell lysates, cells were washed 2× in PBS and lysed in ice-cold RIPA buffer (50 mM Tris-HCl, pH 8.0, 150 mM NaCl, 1% Triton X-100, 0.1% SDS, and 0.5% sodium deoxycholate) in the presence of protease and phosphatase inhibitors (catalog 78440; Pierce/Thermo Fisher). RIPA lysates were sonicated at 20% amplitude for 5 cycles of 2 s on/2 s off on ice using a sonic dismembrator (QSonica, LLC). Alternatively, a postnuclear lysate was obtained by lysing fresh cell pellets in 50 mM Tris, pH 8.0, 150 mM NaCl, 0.5% Triton X-100 on ice for 15 min followed by centrifugation at 14,000 rpm for 10 min at 4°C. Total protein was measured by BCA assay (catalog 23222; Pierce/Thermo Fisher) and reduced immunoblot samples were prepared with 4× sample buffer (125 mM Tris, pH 6.8, 8% LDS, 40% glycerol, Orange G) and 50 mM tris(2-carboxyethyl)phosphine (TCEP) followed by heat-denaturing at 70°C for 15 min. TCEP was omitted for nonreduced samples. Typically, samples of equal protein were run on a range of Bio-Rad TGX minigels and transferred to 0.2 µm nitrocellulose membranes using the Bio-Rad Trans-blot Turbo system. Membranes were blocked with LiCor Odyssey blocking buffer for 1 h at room temperature followed by incubation with primary antibody (diluted in 1:1 TBST/blocking buffer) overnight at 4°C with gentle rocking. HRP-conjugated secondary antibodies (The Jackson Laboratory or Cell Signaling Technologies, CST) diluted in 5% milk/TBST or LiCor fluorescent secondaries diluted in 1:1 TBST/blocking buffer were used. West Dura or West Femto (Pierce) substrate was used for chemiluminescent detection. Blots were imaged using an Odyssey Fc (LiCor) and analyzed using Image Studio software (version 3.1) for densitometry analysis. Detailed information about the anti-PGRN primary antibodies used in this study can be found in Table 3. The following additional primary antibodies were used for immunoblot: neurotensin III/sortilin (1:1000; BD Biosciences, catalog 612101, RRID:AB_399472), LAMP1 (1:5000; CST, catalog 9091), cathepsin D (1:1000; Santa Cruz Biotechnology, catalog sc-6486, RRID:AB_637896), Rab5 (1:1000; CST, catalog 2143, RRID:AB_823625), RCAS1 (1:1000; CST, catalog 12290, RRID:), calnexin (1:1000; CST, catalog 2433, RRID:AB_2243887), COXIV (1:1000; CST, catalog 4850, RRID:AB_2085424), TMEM106B (1:10,000; Pierce/Thermo Fisher, catalog PA5-34353, RRID:AB_2551705), StrepMAB-Immo (1:10,000; IBA, catalog 2-1517-001, RID:AB_513134), or V5 (1:1000; Life Technologies, catalog R960-25, RRID:AB_2556564). Actin (1:10,000; Abcam/Epitomics, catalog ab8227, RRID:AB_2305186), GAPDH (1:5000; Sigma, catalog G8795, RRID:AB_1078991), or tubulin (1:20,000; Abcam/Epitomics, catalog 1878-1, RRID:AB_765089) were used as loading controls.

**Table 3.** List of anti-PGRN primary antibodies used in this study

**Vendor**	**Catalog number/(RRID)**	**Species**	**Clonality**	**Immunogen**	**Antibody stock**	**Immunoblot dilution**	**ICC dilution**
R&D Systems	AF2420/(2114489)	Goat	Poly	aa 18-593	1 µg/µl	1:500-1:1000	1:500
R&D Systems	MAB2420/(2114488)	Mouse	Mono	aa 18-593	1 µg/µl	1:500-1:1000	1:300
Sigma (Atlas)	HPA008763/(1234492)	Rabbit	Poly	aa 128-271	0.1 µg/µl	1:500-1:1000	1:250
Life Technologies	40-3400/(2533461)	Rabbit	Poly	C-terminal protein	0.25 µg/µl	1:500-1:1000	1:50
Novus/SDIX	26320002/(2114484)	Rabbit	Poly	aa 289-426	0.5 µg/µl	1:500	1:250
Abcam/Epitomics	ab108608/EPR3781/(10888818)	Rabbit	Mono	Synthetic protein	0.5 µg/µl	1:500	N/A
ENZO	ALX-804-737-C100/(2052325)	Mouse	Mono	Human PGRN	1 µg/µl	1:1000	N/A
Santa Cruz	(C-11) sc-377036/(Not listed)	Mouse	Mono	aa 21-320	0.2 µg/µl	1:250	1:50
GenScript	Linker-3 (custom)/(n/a)	Rabbit	Poly	aa 187-200 LAKKLPAQRTNRAVC	0.18 µg/µl	1:10,000	N/A
Custom	4C1 (custom)/(n/a)	Mouse	Mono	Human C-TAP PGRN	Unknown	1:50	N/A
LS BIO	LS-C154960/(Not listed)	Goat	Poly	aa 248-259	0.5 µg/µl	1:500	1:50
Adipogen	AG-25A-0090-C100/(249045)	Rabbit	Poly	Human GRN C	1 µg/µl	1:1000	1:250

aa = amino acid, ICC = immunocytochemistry

### Tissue lysis and immunoblotting

Frozen brain tissue from human control (three males and two females) and FTD-*GRN* (two males and three females) cases (Table 4) or mice (of either sex) was first ground under liquid nitrogen to create a uniform powder. Human brain tissue was obtained from Brodmann area 9 of the frontal cortex, an area commonly affected in FTD. Control brains were cognitively normal at autopsy and did not have neuropathological features for major markers of neurodegenerative pathologies including Aβ, tau, α-synuclein, ubiquitin, p62, or TDP-43. FTD-*GRN* cases were classified based on DNA sequencing to harbor a known pathogenic GRN mutation and to have neuropathology consistent with a diagnosis of FLTD using standard measures including staining for p62, ubiquitin, and p-TDP43 (Mackenzie and Neumann, 2017). Roughly equal weights of frozen powder were extracted with the cytoplasmic extraction buffer (CEB) from the Pierce Subcellular Fractionation kit using a Dounce homogenizer according to the manufacturer’s protocol. Homogenates were centrifuged at 500 × *g* for 10 min and the supernatant was saved for total protein analysis (BCA) and SDS-PAGE or ELISA (see below). The remaining pellets were further extracted in 8 M urea to obtain an insoluble protein fraction. For SDS-PAGE, 50 µg of protein was separated on 12 well, 12% Bis-Tris gels (Bio-Rad or Genscript) and transferred to 0.2 µm nitrocellulose membranes in MES buffer followed by immunoblotting as described above. to detect mature GRNs, the membrane was cut at ∼15 kDa, and the bottom was incubated separately with GRN-detecting antibodies to avoid visualization of nonspecific protein bands that migrate at higher MWs.

**Table 4.** Case details of human subjects. PMI = postmortem interval

**Primary neuropathologic diagnosis**	**Sex**	**Age at death (years)**	**PMI (h)**	**Disease duration (years)**
Control	F	60	8	-
Control	F	57	17	-
Control	M	59	6	-
Control	M	65	-	-
Control	M	70	4.5	-
FTD-*GRN*	M	63	-	5
FTD-*GRN*	M	71	15.5	4.5
FTD-*GRN*	F	61	6	6
FTD-*GRN*	F	61	7	6
FTD-*GRN*	F	62	11.5	10
**Group**	**Sex (M/F)**	**Mean ± SEM**	**Mean ± SEM**	**Mean ± SEM**
Control	5 (3/2)	62.2 ± 2.36	8.9 ± 2.80	-
FTD-*GRN*	5 (2/3)	63.6 ± 1.89	10.0 ± 2.19	6.3 ± 0.97

### Protein deglycosylation

Samples of postnuclear lysates from HEK293T cells expressing GRN(+linker) constructs were incubated overnight at 37°C with native PNGase F (New England Biolabs; catalog P0704S) or mock treatment according to the manufacturer’s instructions.

### Secreted PGRN and GRN measurements

HEK-PGRN cells were plated in 6-cm dishes in complete DMEM media (with serum). When the cells were ∼80% confluent, the media were removed, the cells were washed 2× with PBS, and serum-free Opti-MEM media (Life Technologies) was added. After 24 h, the media were removed and immediately centrifuged at 6000 rpm for 5 min to remove any cell debris. The supernatants were directly applied to a 3-kDa MW cutoff spin filter and centrifuged to obtain a 10× concentrated sample (500-50 µl in an Amicon Ultra; Millipore) or 30× concentrated sample (5 ml to ∼160 µl in a Vivaspin 6; GE Healthcare). The resulting concentrates were collected and mixed with 4× sample buffer as above. A total of 20 µl of each media sample was loaded onto a gel for SDS-PAGE followed by transfer to nitrocellulose membranes for total protein stain (REVERT; LiCor) or immunoblot analysis.

### PGRN pulse-chase assay

HAP1 PGRN KO cells were plated in a 12-well dish. The following day, the cells were treated (pulse) with recombinant PGRN diluted in the media for 24 h as indicated. After the 24-h pulse, the media were removed, cells were washed 2× with PBS, and fresh media without recombinant PGRN was applied (chase). Cells were harvested at the indicated time points for immunoblot or immunocytochemistry.

### Lysosome density gradient centrifugation

Approximately 200 mg of fresh HeLa cells were fractionated using the Lysosome Enrichment kit for Tissue and Cultured Cells (Thermo Scientific/Pierce #89839) according to the manufacturer’s protocol. Briefly, the cells were vortexed in 800 µl of enrichment buffer A for 5 s followed by incubation on ice for 2 min. The cell suspension was then transferred to a Dounce homogenizer and homogenized on ice using 70-80 strokes. Next, the lysate was transferred to a 2-ml tube and 800 µl of enrichment buffer B was added. The lysate was inverted several times to mix. The lysate was then centrifuged at 500 × *g* for 10 min at 4°C and the supernatant was collected and transferred to a new tube on ice until needed. Next, in an ultracentrifuge tube, a discontinuous density gradient was prepared by carefully overlaying the prepared OptiPrep Gradients in descending concentrations as follows: 30%, 27%, 23%, 20%, and 17%. The prepared cell extract was mixed with the OptiPrep Cell Separation Media to make a final concentration of 15% OptiPrep Media (1500 µl extract + 500 µl OptiPrep). The 15% OptiPrep sample was then overlaid on top of the gradient and centrifuged at 33,000 rpm for 2 h at 4°C in a Beckman Ti41 swinging-bucket rotor. After centrifugation, 500 µl aliquots of the gradient were carefully removed (12 fractions total) from the top down and used for immunoblot analysis of organelle separation. The top 2 ml of the centrifuged gradient (fractions 1-4) contained the enriched lysosomes.

### Fluorescent immunolabeleing of cells and tissue

HeLa or HAP1 cells were fixed with 4% paraformaldehyde for 30 min at room temperature. Cells were then permeabilized and blocked with 3% BSA + 0.05% saponin in PBS for 1 h at room temperature. Next, primary antibodies were diluted in blocking buffer and incubated overnight at 4°C. Alternatively, some cells were fixed with 4% PFA for 15 min at room temperature followed by permeabilization with ice-cold methanol for 10 min. After blocking with 0.1% BSA in PBS, cells were incubated with primary antibodies overnight at 4°C in blocking buffer. PGRN primary antibodies and their concentrations used are listed in Table 3. Additional primary antibodies used are as follows: LAMP1 (CST; 1:2000), Rab5 (CST; 1:200), RCAS1 (CST; 1:200), calnexin (CST; 1:50), and COXIV (CST; 1:250). After washing with PBS, cells were incubated in secondary antibodies conjugated to Cy5 (1:500; Jackson ImmunoResearch) and/or Alexa Fluor 488 (1:500; Life Technologies) for 1 h at room temperature. Slides were mounted using ProLong Gold Antifade Reagent with DAPI (Life Technologies). For immunolabeling of mouse brains, 30 µm free-floating sections were used. Briefly, brains were harvested and drop-fixed in 4% PFA for 2 d, and then transferred to 30% sucrose until saturation. Sectioning was performed on a freezing sliding microtome and free-floating sections stored in cryoprotectant solution. For immunostaining, sections were thoroughly washed in PBS and blocked for 1 h in 5% normal goat serum in PBS, with 0.5% Triton X-100 for permeabilization. Sections were then incubated with the following primary antibodies diluted in blocking solution overnight at 4°C: V5 (Life Technologies, 1:1000) and NeuN (EMD Millipore; 1:500). After washing in PBS, sections were incubated with secondary antibodies conjugated to Cy-3 (Jackson ImmunoResearch; 1:500) and Alexa Fluor 488 (Life Technologies, 1:500) diluted in blocking solution with addition of DAPI (Life Technologies, 1:1000) for 1 h at room temperature. Finally, sections were washed in PBS and mounted using Vectashield Hard Set (Vector Laboratories). Images were captured at room temperature with an EVOS FL Cell Imaging System (20× objective; Life Technologies) and postprocessed in Adobe PhotoShop CS5. For Super Resolution Microscopy, images (z-stack images encompassing GRN and LAMP1 signal, typically 30-40 planes, 125-nm step size) were taken at room temperature using a GE Delta Vision OMX Blaze with 3D Structure Illumination Microscopy (SIM; 60× NA 1.42 objective; GE Healthcare Life Sciences). Raw structured illumination data were reconstructed using DeltaVision’s SoftWorx application, and the resulting high resolution z-stacks were visualized and processed using Fiji and Imaris v8.2 software (Bitplane).

### PGRN ELISA

Human Progranulin DuoSet ELISA reagents were from R&D Systems and the ELISA was conducted according to the manufacturer’s protocol. Cell culture media samples were pre-cleared of cell debris by spinning for 5 min at 6000 rpm at 4°C and diluted 1:4 or 1:5 in 1× Reagent Diluent #2. Brain lysates were diluted 1:5 in 1× Reagent Diluent #2. All ELISA measurements were done in duplicate and fell within the standard curve generated by the provided recombinant PGRN standard. PGRN measurements were normalized to the total protein in the lysate and compared to the indicated control.

### Cathepsin L studies

Recombinant human cathepsin L was from R&D Systems (#952-CY). For *in vitro* cleavage assays, recombinant cathepsin L was incubated with recombinant human C-TAP PGRN at a ratio of 1:10 (enzyme:substrate, typically 200:2000 ng) in assay buffer [50 mM MES, 5 mM DTT, 1 mM EDTA, 0.005% (w/v) Brij-35, pH 6.0] at 37°C for the indicated times. At each time point, an aliquot of the mixture was removed and mixed with 4× sample buffer for immunoblot analysis. For cell culture inhibitor studies, HAP1 wild-type (WT) cells were plated in 12-well plates. The following day, the cells were treated with the cathepsin L inhibitor II (Z-FY-CHO; Calbiochem) in the media at the concentrations indicated. HAP1 WT cells were harvested after 40 h for immunoblot analysis. Cells were lysed and analyzed by immunoblot as described above.

### Human adeno-associated virus serotype 2/1 (AAV2/1)/1-PGRN injections

All animal procedures were performed in accordance with the Emory University animal care committee's regulations. We performed somatic brain transgenesis (SBT) to overexpress eGFP or human N-TAP PGRN in *Grn* KO mice of either sex. Briefly, recombinant AAV2/1 encoding eGFP or human N-TAP PGRN were generated and neonatal P0 injections of rAAV2/1 were performed (Chakrabarty et al., 2013).

## Results

### Expression of recombinant GRN proteins and identification of antibodies that detect GRNs

**Figure 1.**
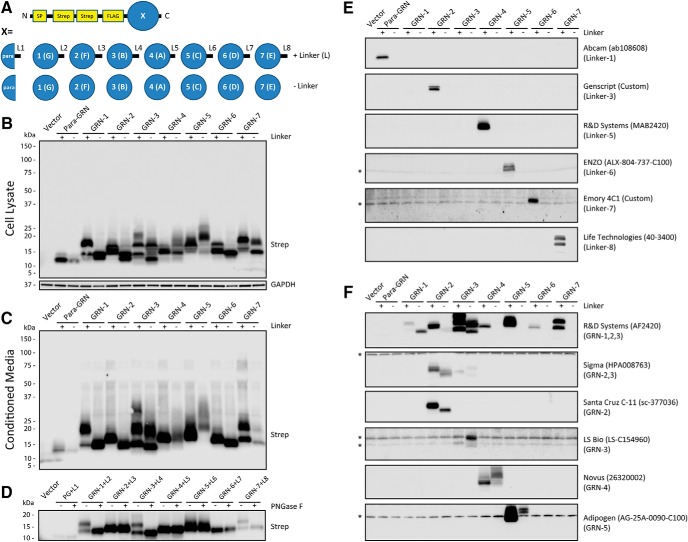
Expression of recombinant human GRNs and identification of antibodies that detect GRNs. ***A***, Schematic of human GRN expression constructs. Human GRN sequences (Table 2), with and without adjacent C-terminal linker regions, were synthesized to include the N-terminal PGRN signal peptide (SP), followed by twin-Strep (SAWSHPQFEK) tags and a single FLAG (DYKDDDDK) tag. Throughout the manuscript, individual GRNs are referred to by their numerical sequential designation (i.e., GRN-1, GRN-2, etc.), which correspond to their original alphabetical designation (i.e., GRN-G, GRN-F, etc.). ***B***, ***C***, HEK293T cells were transfected with the human GRN constructs and 48 h later either (***B***) whole-cell lysates or (***C***) conditioned media were analyzed by immunoblot for protein expression using the StrepMAB-immo antibody. ***D***, Cell lysates from GRN(+linker) overexpressing cells were mock (-) or PNGase F (+) treated to detect glycosylated proteins. ***E***, ***F***, GRN-expressing HEK293T cell lysates were analyzed by immunoblot to identify PGRN antibodies that either (***E***) detect single linker regions of PGRN or (***F***) detect GRNs. The specific PGRN linker or GRN(s) detected are listed under each antibody. Detailed information about the PGRN antibodies screened can be found in Table 3. Asterisks (*) denote nonspecific protein bands.

The lack of tools to study endogenous GRNs has limited the field’s ability to study the normal functions of PGRN and to determine how loss of PGRN leads to lysosome dysfunction and neurodegeneration. To overcome this gap, we developed a system to selectively express each human GRN in mammalian cells. This system enabled us to screen anti-human PGRN antibodies by immunoblot to determine which region(s) of PGRN they detect. First, we generated expression constructs for each of the human GRN proteins: para-GRN, GRN-1, GRN-2, GRN-3, GRN-4, GRN-5, GRN-6, and GRN-7, with (+) and without (-) their endogenous C-terminal linker regions (Fig. 1*A*; Table 2; Materials and Methods). At the N terminus, each GRN construct was engineered to contain the authentic PGRN signal peptide to ensure proper routing into the secretory pathway, followed by twin-Strep tags and a FLAG tag, to facilitate purification and detection. Next, we transfected the GRN constructs into HEK293T cells and were able to detect all of the expressed GRN proteins in cell lysates and conditioned media, indicating GRNs are properly synthesized and secreted (Fig. 1*B*,*C*). Some GRNs migrated as multiple bands, suggesting post-translational glycosylation. To confirm this, we incubated GRN(+linker) overexpressing cell lysates with or without PNGase F and detected proteins by immunoblot (Fig. 1*D*). We found that several GRNs or linker regions, including GRN-1(+L2), GRN-3(+L4), and GRN-7(+L8), collapsed to a single band after PNGase treatment, indicating these regions are glycosylated, consistent with published N-linked glycosylation sites (Songsrirote et al., 2010). Next, we used our expression system to screen a panel of commercial and in-house polyclonal and monoclonal antibodies developed against recombinant full-length PGRN or peptides using immunoblot (Table 3). Under reducing conditions, we found that many of the PGRN antibodies detect a single linker region of PGRN, suggesting that these unstructured regions of PGRN may be immuno-dominant epitopes (Fig. 1*E*). Encouragingly, we also identified multiple antibodies that detect one or more specific GRNs (Fig. 1*F*). These included the AF2420 (R&D Systems) polyclonal antibody, which detects GRN-1, GRN-2 (weakly), and GRN-3 and antibodies that detect GRN-2 (Sigma, Santa Cruz C-11), GRN-3 (LS Bio, Sigma weakly), GRN-4 (Novus), and GRN-5 (Adipogen). Notably, the AF2420 antibody detects nearly all linker regions of PGRN, in addition to multiple GRNs, making it a potentially useful tool for dissecting intermediate PGRN cleavage products in combination with other linker-specific antibodies.

### Endogenous, intracellular GRNs are present in various cell types

**Figure 2.**
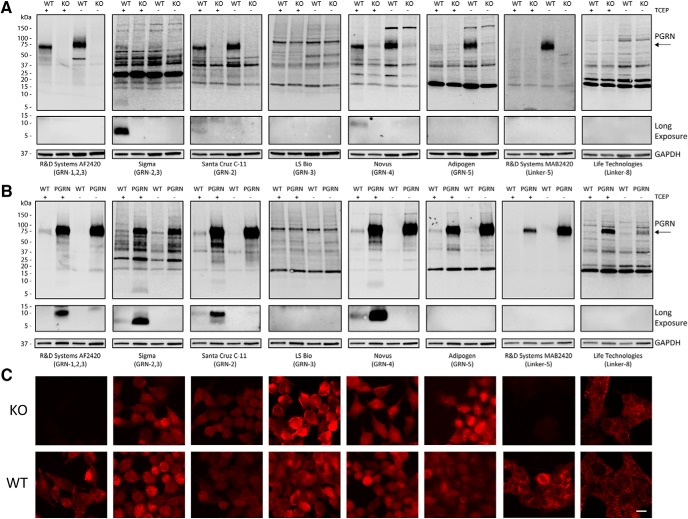
Specificity of PGRN antibodies and detection of multiple endogenous GRNs. ***A***, Whole-cell lysates from HAP1 WT and HAP1 PGRN KO cells, either reduced (+TCEP) or nonreduced (-TCEP), were analyzed for PGRN antibody specificity by immunoblot. The bottoms of the blots (<15 kDa) were exposed longer to reveal levels of endogenous GRNs. ***B***, Whole-cell lysates from HEK293T WT and N-TAP PGRN stable overexpressing (PGRN) cells were analyzed as in A by immunoblot. ***C***, Representative immunofluorescent images of HAP1 PGRN KO and WT cells stained with the corresponding PGRN antibodies as in A and B. Scale bar, 10 µm.

Next, we determined if any of the PGRN antibodies could detect endogenous GRNs in cell lysates by immunoblot. To test antibody specificity, we knocked out *GRN* in a near-haploid human cell line (Essletzbichler et al., 2014) using CRISPR-Cas9 to generate the HAP1 PGRN KO cell line. Then, we compared antibody reactivity in whole-cell lysates from HAP1 WT and PGRN KO lines. Most antibodies detected endogenous, full-length PGRN in HAP1 WT lysates at the expected MW of ∼75 kDa (Fig. 2*A*). In addition, we also observed multiple immunoreactive bands in the 15- to 75-kDa range that were present in both WT and PGRN KO cells, indicating they are nonspecific. This result highlights the importance of extensive antibody validation (Uhlen et al., 2016) and is especially important for PGRN, because nonspecific bands could be misinterpreted as PGRN cleavage products. Two antibodies (Sigma and Novus) detected specific bands at lower MWs (<15 kDa) that correspond to the expected size of individual mature GRN proteins (Fig. 2*A*). Next, we screened the same panel of PGRN antibodies against immunoblots of whole-cell lysates from HEK293T cells stably overexpressing human PGRN (HEK-PGRN). Here, we identified multiple antibodies (R&D AF2420, Sigma, Santa Cruz C-11, Novus) that detected specific bands at the expected MWs for GRNs that were increased in the HEK-PGRN lysates compared to HEK-WT lysates (Fig. 2*B*). GRNs were only detected in the reduced samples (+TCEP), whereas full-length PGRN could often be detected in reduced or nonreduced samples. We did not detect any endogenous GRN bands <15 kDa in the HEK-PGRN lysates using the antibodies specific for GRN-3 (LS Bio) or GRN-5 (Adipogen; Fig. 2*B*). Together, these data demonstrate that multiple GRN proteins, specifically GRN-2 and GRN-4, are endogenously present inside cells. Finally, we assessed the specificity of the PGRN antibodies using fluorescent immunocytochemistry in HAP1 WT and PGRN KO cells. We found that only the R&D MAB2420 and R&D AF2420 antibodies were specific for PGRN/GRN immunolabeling in these cells (Fig. 2*C*). The nonspecific staining of the other PGRN antibodies mirrors the nonspecificity that we observed by immunoblots in Figure 2*A*,*B*.

**Figure 3.**
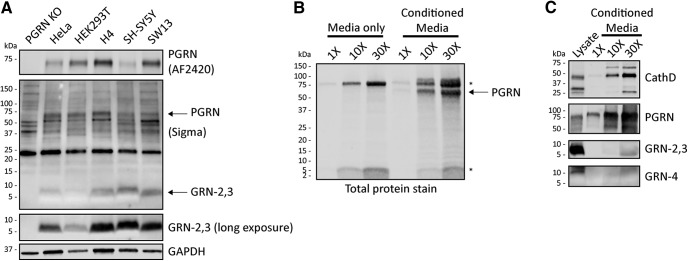
Endogenous GRNs are found intracellularly in a variety of cell types. ***A***, Whole-cell lysates (30 µg total protein) from commonly used cell types were analyzed for intracellular PGRN and GRN-2,3 by immunoblot. The entire Sigma immunoblot is shown to highlight the prominent nonspecific bands detected by the antibody (see PGRN KO lane). ***B***, Serum-free Opti-MEM media alone or conditioned media from HEK-PGRN cells grown for 24 h was concentrated, run on SDS-PAGE, and transferred to nitrocellulose membrane. Total proteins were visualized with REVERT total protein stain (LiCor) to ensure retention of low MW proteins. The bands designated with asterisks (*) indicate proteins found in Opti-MEM media alone whereas the arrow points to secreted N-TAP PGRN. ***C***, Concentrated serum-free conditioned media from HEK-PGRN cells was analyzed for secreted cathepsin D, PGRN (R&D AF2420), and GRNs by immunoblot. All images are representative of at least two independent experiments.

Based on our observations, the Sigma antibody was the most sensitive and reliable for detection of human GRN by immunoblot. This antibody strongly recognizes GRN-2, with weaker reactivity to GRN-3 (referred to as GRN-2,3; Fig. 1*F*), thus we cannot conclusively say whether it detects endogenous GRN-2, GRN-3, or both in cell lysates. Nevertheless, we used this antibody to further study endogenous GRN-2,3 expression and production. We found that GRN-2,3 could be detected at steady-state levels in all human cell lines tested, notably with the highest levels in H4 (neuroglioma) and SH-SY5Y (neuroblastoma) cell lines (Fig. 3*A*). To determine if GRNs can be detected extracellular in the media of cultured cells, we grew HEK-PGRN cells in serum-free media (Opti-MEM) for 24 h, concentrated the media in a centrifugal spin filter (3-kDa MW cutoff), and analyzed the concentrate by total protein stain and immunoblot. As a control, we also concentrated Opti-MEM media alone. We detected a ∼5-kDa protein band in both the conditioned media and media alone that increased in intensity with concentration (Fig. 3*B*). This protein is likely insulin (MW, 5.8 kDa), which is a principle component of Opti-MEM media, and confirmed that low MW proteins similar to GRNs in size were enriched in the concentrate. By immunoblot, we verified robust levels of secreted PGRN and cathepsin D, which served as a positive control for a secreted lysosomal protein, in the concentrated media. Conversely, GRNs (-2,3; Sigma and -4; Novus) were barely detectable, even in the 30× concentrated media sample (Fig. 3*C*). These results indicate that these GRNs do not appear to be constitutively secreted or generated from secreted PGRN in cultured cells.

### GRNs are stable lysosomal proteins

**Figure 4.**
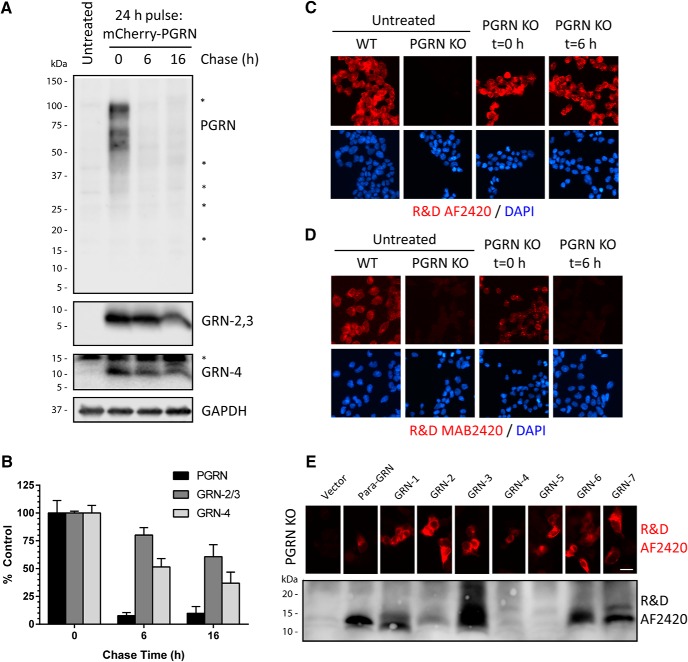
Endocytosed PGRN is rapidly processed into stable, mature GRNs. ***A***, HAP1 PGRN KO cells were pulsed with mCherry-PGRN (5 µg/ml) in the media for 24 h and then chased with fresh media without PGRN for various lengths of time. Lysates were analyzed for PGRN (R&D AF2420), GRN-2,3 (Sigma), and GRN-4 (Novus) by immunoblot. Equal volumes of lysates were run for each time point to account for differences in cell growth and measurements were normalized to untreated control (PGRN KO) background signal. Asterisks (*) denote nonspecific proteins. ***B***, Quantification of PGRN and GRNs from A. Data represent mean ± SEM of three independent replicates for each time point normalized to *t* = 0 h control. ***C***, ***D***, HAP1 PGRN KO cells pulsed with mCherry-PGRN (5 µg/ml) for 24 h (*t* = 0 h) and then chased with fresh media for 6 h (*t* = 6 h) were immunolabeled with either (***C***) R&D AF2420 (recognizes multiple PGRN linkers and GRNs) or (***D***) R&D MAB2420 (recognizes only linker-5 of PGRN). Untreated HAP1 WT and PGRN KO cells are shown as specificity controls for antibody labeling. ***E***, top, HAP1 PGRN KO cells were transfected with GRN(-linker) constructs and immunolabeled with R&D AF2420. Scale bar, 5 µm. Bottom, nonreduced (-TCEP) lysates from HEK293T cells transfected with GRN(-linker) constructs were immunoblotted with R&D AF2420.

To monitor the production, stability, and localization of GRNs inside cells, we developed a PGRN pulse-chase assay based on the observation that application of purified extracellular PGRN was rapidly endocytosed by many cell lines. HAP1 GRN KO cells were treated (pulsed) with human mCherry-PGRN for 24 h and then chased with fresh media without PGRN for varying lengths of time. The levels of PGRN (R&D AF2420), GRN-2,3 (Sigma), and GRN-4 (Novus) were measured at each time point by immunoblot. We found that after a 24 h pulse of mCherry-PGRN, robust intracellular PGRN and GRN signals were observed by immunoblot (Fig. 4*A*). After a 6-h chase, the PGRN signal was virtually undetectable (Fig. 4*A*,*B*). Strikingly, a strong signal for GRN-2,3 and GRN-4 was still detected out to 16 h (Fig. 4*A*,*B*). These data indicate that endocytosed PGRN is rapidly processed into mature, stable GRNs. Similar results were obtained using purified N-TAP PGRN in the pulse-chase assay (data not shown). It is worth noting that we did not observe any accumulation of intermediate PGRN fragments in the ∼15- to 60-kDa range at any time point during the pulse-chase assay using the R&D AF2420 antibody which detects multiple linker regions of PGRN (Fig. 4*A*).

Next, we immunostained PGRN KO cells pulse-chased with mCherry-PGRN (24-h pulse/6-h chase). Following a 6-h chase, the R&D AF2420 antibody still gave a robust immunofluorescent signal (Fig. 4*C*). At this time point (*t* = 6 h) we only detect GRNs, not PGRN, by immunoblot (Fig. 4*A*). As a control, we stained another set of cells with the R&D MAB2420 antibody which is linker specific and only recognizes full-length PGRN. R&D MAB2420 staining revealed punctate labeling after a 24 h pulse, but the signal was eliminated after the 6-h chase, indicating that full-length, endocytosed PGRN had been metabolized. These results demonstrate that the R&D AF2420 antibody can be used for immunofluorescent labeling of GRNs. To confirm this, we expressed every GRN(-linker) construct in PGRN KO cells for 24 h and immunostained them with the AF2420 antibody (Fig. 4*E*). Surprisingly, the antibody detected every expressed GRN protein. We then immunoblotted the GRN(-linker) overexpressed cell lysates from HEK293T cells under nonreducing conditions (-TCEP) and found that the AF2420 antibody now recognized nearly every GRN. These results indicate that the AF2420 polyclonal antibody may detect a conserved GRN motif under native-like conditions. In addition, the immunofluorescence data confirm processing of PGRN into stable GRNs and validate a useful tool for studying PGRN/GRN localization inside cells.

**Figure 5.**
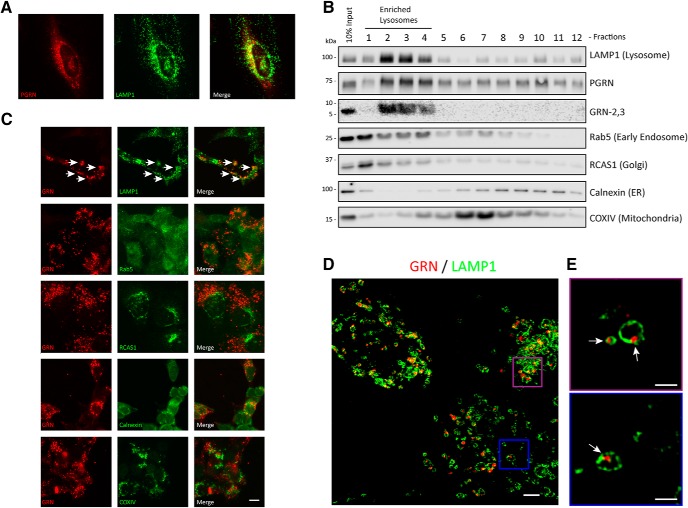
GRNs localize to lysosomes. ***A***, HeLa cells were double immunolabeled with PGRN (R&D AF2420) and LAMP1 antibodies. ***B***, Endogenous HeLa proteins were separated by density-based gradient centrifugation and individual fractions (1-12) were analyzed for PGRN (R&D AF2420), GRN-2,3 (Sigma), and organelle markers by immunoblot. Results are representative of two independent experiments. ***C***, HAP1 PGRN KO cells were pulse-chased (24-h pulse/6-h chase) with mCherry-PGRN (5 µg/ml) and then double immunolabeled for GRNs (R&D AF2420) and organelle markers. White arrows indicate colocalization of GRNs and LAMP1. Scale bar, 10 µm. ***D***, HAP1 GRN KO cells were pulse-chased as in ***C***, double immunolabeled for GRNs (R&D AF2420) and LAMP1, and imaged using Super Resolution microscopy. All z-stacks included. Scale bar, 2 µm. ***E***, Magnified regions of D show that GRNs are mostly found associated with the inner membrane of LAMP1-positive lysosomes (white arrows). Select z-stacks of ***D*** shown for clarity. Scale bar, 1 µm.

Extracellular or newly synthesized PGRN can be routed to the lysosome via multiple mechanisms (Hu et al., 2010; Zhou et al., 2015) where it colocalizes with the lysosomal marker, LAMP1. In HeLa cells, we observed significant overlap of LAMP1 and PGRN (R&D AF2420) costaining (Fig. 5*A*), as has been previously reported (Chen-Plotkin et al., 2012). However, it has been unclear whether full-length PGRN or GRNs are the main species localized to lysosomes. To address this, we used the validated antibodies that detect GRNs to explore the production and localization of GRNs inside cells. First, we performed density-based gradient centrifugation on HeLa lysates to determine where endogenous PGRN and GRN-2,3 fractionate based on organelle-specific proteins (Fig. 5*B*). PGRN was found throughout all of the fractions, which indicates its presence in the endoplasmic reticulum (ER), Golgi apparatus, and endosomes as previously reported (Ryan et al., 2009; Almeida et al., 2011; Capell et al., 2011). Alternatively, GRN-2,3 were exclusively concentrated in fractions that were most enriched for LAMP1, indicating their likely localization to lysosomes. Next, we assessed colocalization of GRNs with organelle markers in PGRN KO cells pulse-chased with PGRN (24-h pulse/6-h chase) using double-label immunofluorescence. Again, GRNs (as assessed by R&D AF2420) showed significant colocalization with the lysosomal marker LAMP1 and did not overlap with markers for early endosomes (Rab5), the Golgi apparatus (RCAS1), the ER (calnexin), or mitochondria (COX IV; Fig. 5*C*). To analyze the localization of GRNs to lysosomes in more detail, we coupled our PGRN pulse-chase assay with super-resolution microscopy (Fig. 5*D*). We found that GRNs were often associated with the inner leaflet of LAMP-1-positive vesicles that are likely lysosomes, suggesting that a portion of GRNs may be associated with the lysosome membrane and/or membrane-bound proteins (Fig. 5*E*). Taken together, these data demonstrate that GRNs, rather than PGRN, are the predominant stable species present in lysosomes.

### GRN levels are regulated by expression of SORT1 or TMEM106B

**Figure 6.**
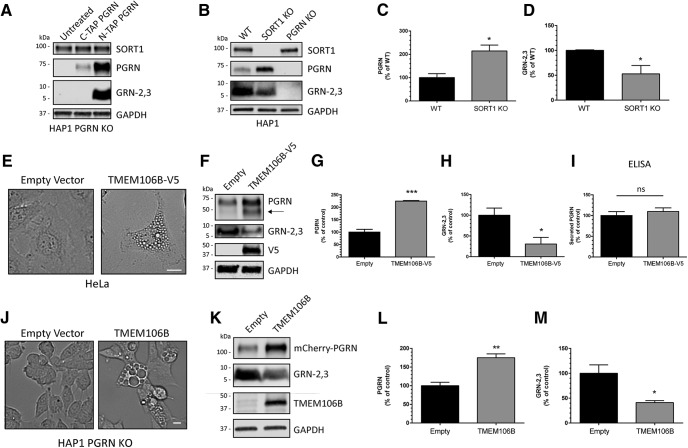
GRN levels are regulated by SORT1 and TMEM106B expression. ***A***, HAP1 PGRN KO cells were treated with C-TAP PGRN or N-TAP PGRN (5 µg/ml) for 24 h and lysates were analyzed for PGRN and GRN-2,3 by immunoblot. ***B***, HAP1 WT, SORT1 KO, and PGRN KO cell lysates were analyzed for endogenous levels of SORT1, PGRN, and GRN-2,3 by immunoblot. ***C***, ***D***, Quantification of (***C***) PGRN and (***D***) GRN-2,3 from the experiment in ***B***. ***E***, Overexpression of TMEM106B in HeLa cells for 48-h results in the formation of large vacuoles. Scale bar, 20 µm. ***F***, HeLa cells were transfected with empty vector or TMEM106B for 48 h and lysates were analyzed for PGRN and GRN-2,3 by immunoblot. ***G-I***, Quantification of (***G***) intracellular PGRN, (***H***) intracellular GRN-2,3, and (***I***) secreted PGRN (by ELISA) from the experiment in ***F***. Arrow in ***F*** denotes endogenous, intermediate PGRN cleavage product. ***J***, Overexpression of TMEM106B for 24 h in HAP1 PGRN KO cells results in the formation of large vacuoles. Scale bar, 10 µm. ***K***, HAP1 PGRN KO cells were transfected with TMEM106B for 24 h and then treated with mCherry-PGRN (5 µg/ml) for an additional 24 h. Lysates were analyzed for PGRN and GRN-2,3 by immunoblot. ***L***, ***M***, Quantification of (***L***) PGRN and (***M***) GRN-2,3 from the experiment in ***K***. For all immunoblots, PGRN and GRN-2,3 were detected with R&D AF2420 and Sigma antibodies, respectively. All immunoblot images are representative of at least three independent experiments and quantitative data are presented as mean ± SEM of three independent experiments; *differs from control *p* < 0.05; **p < 0.01; ***p < 0.001; ns = not significant.

PGRN levels have been linked to expression of several genes including *SORT1*, encoding the membrane receptor sortilin (Carrasquillo et al., 2010; Hu et al., 2010), and *TMEM106B*, encoding TMEM106B (Van Deerlin et al., 2010; Finch et al., 2011; Nicholson et al., 2013). Thus, we reasoned that altered expression of these proteins would affect the production of intracellular GRNs as well. To address this, we first determined if expression of sortilin, which facilitates endocytosis and trafficking of PGRN to the lysosome (Carrasquillo et al., 2010; Hu et al., 2010), influences GRN levels. We treated PGRN KO cells with PGRN tagged at either the C terminus (C-TAP) (Chen et al., 2013), which disrupts sortilin binding (Zheng et al., 2011) or the N terminus (N-TAP), which preserves sortilin binding, and measured intracellular PGRN and GRN-2,3 by immunoblot. We found that C-TAP PGRN was not as efficiently endocytosed and processed into GRNs compared to N-TAP PGRN (Fig. 6*A*), indicating that sortilin plays a major role in the uptake of extracellular PGRN in the HAP1 cell line. Next, we generated HAP1 SORT1 KO cells using CRISPR/Cas9 technology and verified they do not produce sortilin by immunoblot (Fig. 6*B*). SORT1 KO cells had significantly increased levels of PGRN (Fig. 6*C*) and significantly reduced levels of GRN-2,3 (Fig. 6*D*) compared to WT cells. Thus, disruption of the SORT1/PGRN axis appears to alter intracellular PGRN trafficking to the lysosome, leading to decreased GRN production. Moreover, because genetic deletion of SORT1 did not completely eliminate production of GRN, this data supports that other pathways exist to traffic PGRN to the lysosome as recently reported (Zhou et al., 2015).

Next, we tested if overexpression of the FTD-*GRN* modifier TMEM106B affects production of GRNs. TMEM106B is a neuronal, lysosomal protein and its overexpression in cells causes lysosomal dysfunction and increased intracellular PGRN levels (Chen-Plotkin et al., 2012; Brady et al., 2013; Stagi et al., 2014; Busch et al., 2016). Notably, increased levels of TMEM106B have been found in FTD patient brains (Chen-Plotkin et al., 2012). Here, we transfected TMEM106B into HeLa cells and measured PGRN and GRN-2,3 levels by immunoblot after 48 h. TMEM106B expression resulted in the accumulation of large vacuoles indicative of impaired lysosomal acidification and function (Busch et al., 2016; Fig. 6*E*) and a significant increase in intracellular PGRN (Fig. 6*F*,*G*). Conversely, GRN-2,3 levels were significantly decreased (Fig. 6*F*,*H*). We did not detect any difference in secreted PGRN levels with TMEM106B expression (Fig. 6*I*), indicating that TMEM106B overexpression likely inhibits intracellular processing of PGRN into GRNs. To assess whether TMEM106B overexpression specifically affects processing of endocytosed PGRN, we transfected PGRN KO cells with TMEM106B for 24 h and then treated them with mCherry-PGRN for an additional 24 h. Once again, TMEM106B overexpression in HAP1 cells resulted in enlarged vacuoles (Fig. 6*J*) and significantly reduced processing of endocytosed PGRN into GRN-2,3 (Fig. 6*K-M*). Together, these data indicate that TMEM106B may increase FTD risk by inhibiting the processing of PGRN into GRNs through lysosome dysfunction or altered trafficking of PGRN.

### The processing of PGRN into GRNs is inhibited by pharmacologic lysosome inhibition and cysteine protease inhibitors

**Figure 7.**
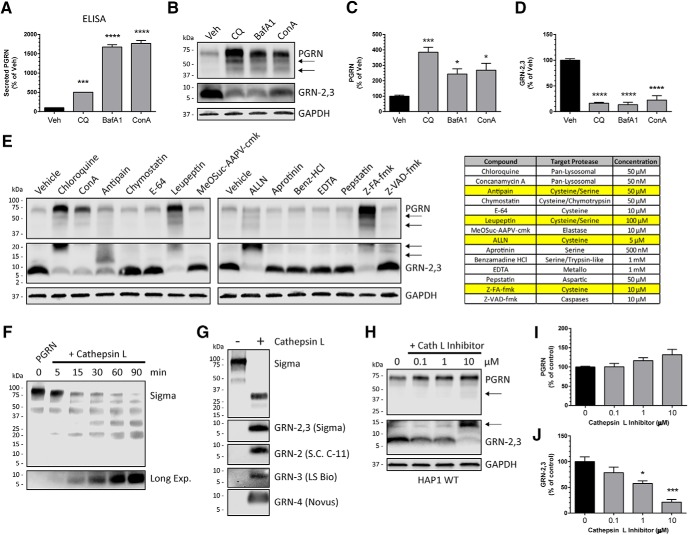
PGRN processing into GRNs is mediated by proper lysosome function and cysteine protease activity. ***A***, HAP1 WT cells were treated for 24 h with the pan-lysosome inhibitors chloroquine (CQ; 50 µM), BafA1 (50 nM), or concanamycin A (ConA; 50 nM) and conditioned media were analyzed for secreted PGRN by ELISA. ***B***, Lysates from HAP1 WT cells treated as in ***A*** were analyzed for PGRN and GRN-2,3 by immunoblot. ***C***, ***D***, Quantification of (***C***) PGRN and (***D***) GRN-2,3 from the experiment in ***B***. ***E***, HAP1 WT cells were treated with the indicated protease inhibitors for 24 h and analyzed for PGRN and GRN-2,3 by immunoblot. The inhibitors, their primary targets, and concentrations used are shown in the table at right. ***F***, Time-dependent cleavage of C-TAP PGRN by recombinant cathepsin L *in vitro*. ***G***, C-TAP PGRN was incubated with or without cathepsin L for 2 h *in vitro* and analyzed for multiple GRNs by immunoblot. ***H***, HAP1 WT cells were treated with increasing concentrations of cathepsin L inhibitor II (Z-FY-CHO) for 40 h and lysates were analyzed for PGRN and GRN-2,3 by immunoblot. ***I***, ***J***, Quantification of (***I***) PGRN and (***J***) GRN-2,3 from the experiment in ***H***. ***B***, ***E***, ***H***, Arrows denote endogenous, intermediate PGRN cleavage products. For all immunoblots, PGRN and GRN-2,3 were detected with R&D AF2420 and Sigma antibodies respectively. All immunoblot images are representative of at least three independent experiments and quantitative data are presented as mean ± SEM of three independent experiments; *differs from control *p* < 0.05; **p < 0.01; ***p < 0.001; ****p < 0.0001.

Because we observed the rapid processing of endocytosed PGRN into GRNs, we reasoned that inhibiting lysosome function would impair GRN production. To determine the effect of pharmacologic lysosome inhibition on GRN levels, we treated HAP1 WT cells with the pan-lysosomal inhibitors chloroquine, BafA1, or concanamycin A1 for 24 h and monitored PGRN and GRN-2,3 levels by immunoblot. These compounds act as lysosome alkalizing agents or inhibitors of the vacuolar-type H+-ATPase (V-ATPase), which is needed for proper lysosome acidification and function. Further, these compounds were previously found to robustly increase intracellular and secreted PGRN (Capell et al., 2011). In our experiments, both secreted PGRN (Fig. 7*A*) and intracellular PGRN (Fig. 7*B*,*C*) were significantly increased by all of the pan-lysosomal inhibitors as expected. In contrast, GRN-2,3 levels were significantly decreased (Fig. 7*B*,*D*), indicating that the processing of PGRN to GRNs was inhibited and depends on the proper acidification and function of lysosomes.

Processing of PGRN into GRNs likely involves one or more lysosomal proteases. To narrow down which class of proteases may be responsible for intracellular PGRN metabolism, we treated HAP1 WT cells with a panel of protease inhibitors and measured PGRN and GRN-2/3 levels by immunoblot. Inhibitors of serine, aspartic, metallo-, or trypsin-like proteases did not affect GRN-2,3 production (Fig. 7*E*). However, multiple inhibitors of cysteine proteases (antipain, leupeptin, ALLN, and Z-FA-fmk) reduced GRN-2,3 levels compared to vehicle-treated control (Fig. 7*E*). One potential candidate lysosomal cysteine protease is cathepsin L, which was reported to cleave mouse PGRN *in vitro* (Park et al., 2011). We found that incubation of cathepsin L and recombinant human PGRN lead to the time-dependent decrease of full-length PGRN and a corresponding increase of GRN-2,3 protein (Fig. 7*F*). Interestingly, the *in vitro* cleavage of PGRN by cathepsin L generated multiple, mature GRNs, including GRN-2, GRN-3, and GRN-4 (Fig. 7*G*). Finally, treatment of HAP1 WT cells with the cathepsin L inhibitor II (Z-FY-CHO; Calbiochem) modestly increased endogenous PGRN (Fig. 7*H*,*I*), and significantly decreased production of GRN-2,3 (Fig. 7*H*,*J*). Overall, these data indicate that properly functioning lysosomes and cysteine proteases in particular contribute to the intracellular processing of PGRN into GRNs.

### Human PGRN is processed into mature GRNs in mouse cells and brain

**Figure 8.**
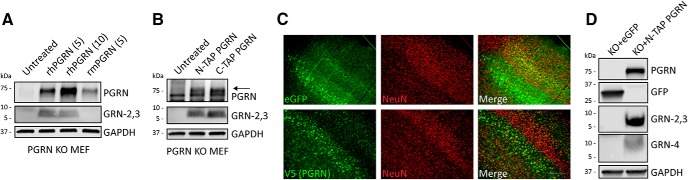
Human PGRN is processed into GRNs in mouse cells and in mouse brain. ***A***, PGRN KO MEF cells were treated with recombinant human PGRN (5 or 10 µg/ml) or recombinant mouse PGRN (5 µg/ml) in the media for 24 h and lysates were analyzed for PGRN (R&D AF2420) and GRN-2,3 (Sigma) by immunoblot. R&D AF2420 displays some cross-reactivity with mouse PGRN. ***B***, PGRN KO MEF cells were treated with N-TAP or C-TAP human PGRN (5 µg/ml) in the media for 24 h and lysates were analyzed for PGRN and GRN-2,3 by immunoblot. ***C***, Cortical sections from three-month-old GRN KO mice injected with AAV2/1-eGFP or AAV2/1-NTAP-PGRN at *P* = 0 show colocalization of eGFP or PGRN (V5) expression with the neuronal marker NeuN. Scale bar, 200 µm. ***D***, Brain tissue lysates from 14-month-old GRN KO mice injected as in C were analyzed for human PGRN (R&D AF2420) and GRNs (-2,3; Sigma and -4; Novus) by immunoblot. Immunoblots in ***A***, ***B*** are representative of two independent experiments each. Images in ***C***, ***D*** are representative of at least three independent eGFP- or PGRN-injected mice.

Because this study focused on screening and validating human-specific PGRN antibodies, we have not yet identified antibodies to detect endogenous mouse GRNs. Nevertheless, we wanted to determine if mouse cells are able to process human PGRN into mature GRNs. To accomplish this goal, we first treated primary PGRN KO MEFs with recombinant human or mouse PGRN for 48 h and measured PGRN and GRN-2,3 levels in the lysates by immunoblot. PGRN KO MEFs efficiently endocytosed human PGRN and generated mature human GRN-2,3 (Fig. 8*A*). The Sigma antibody did not detect any endogenous GRN bands in the PGRN KO MEFs treated with mouse PGRN, confirming that it is specific to human GRNs (Fig. 8*A*). Further, PGRN KO MEFs were able to internalize N-TAP or C-TAP human PGRN and generate GRNs equally well (Fig. 8*B*), indicating that primary MEFs do not require sortilin for PGRN uptake. Next, we asked if human PGRN would be processed into GRNs when expressed in mouse brain. We performed SBT by injecting rAAV2/1 encoding eGFP or human N-TAP PGRN into the cerebral ventricles of newborn (P0) PGRN KO mice. The SBT paradigm leads to transgene expression that is almost exclusively neuronal (Chakrabarty et al., 2013) as assessed by predominant colocalization between eGFP or PGRN (V5) and the neuronal marker NeuN at three months of age (Fig. 8*C*). Mice harvested at 14 months of age robustly expressed human PGRN (R&D AF2420) and produced human GRNs (-2,3; Sigma and -4; Novus) in brain tissue lysates as assessed by immunoblot (Fig. 8*D*). Taken together, these data demonstrate that the generation of GRNs from PGRN, including the required sorting pathways and protease(s), are conserved between humans and mice.

### Multiple GRNs are haploinsufficient in FTD-*GRN* patient fibroblasts and brain

**Figure 9.**
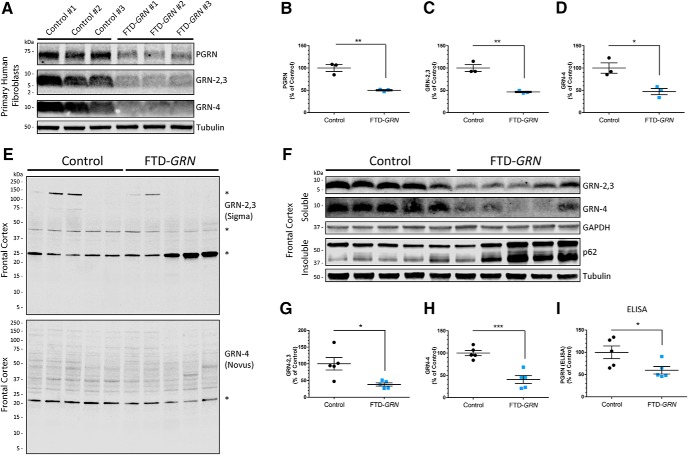
FTD patients with a *GRN* mutation (FTD-*GRN*) are haploinsufficient for multiple GRNs. ***A***, Primary fibroblast lysates from three control and three FTD-*GRN* patients were analyzed for PGRN and GRNs by immunoblot. ***B-D***, Quantification of (***B***) PGRN, (***C***) GRN-2,3 (Sigma), and (***D***) GRN-4 (Novus) from the experiment in ***A***. ***E***, Full-blot images of soluble brain lysates from five control and five FTD-*GRN* patients using Sigma or Novus anti-GRN antibodies showing predominant nonspecific protein detection (denoted by *). ***F***, The same samples from E were run on SDS-PAGE and analyzed for mature GRNs by immunoblot as outlined in Materials and Methods. Immunoblots for insoluble p62 indicate disease-associated pathology in the FTD-*GRN* cases. ***G-I***, Quantification of (***G***) GRN-2,3 (Sigma) and (***H***) GRN-4 (Novus) from the experiment in ***F***. ***I***, Quantification of PGRN in the same soluble brain lysates from F by ELISA. All data represent the mean ± SEM; *differs from control *p* < 0.05; **p < 0.01; ***p < 0.001.

Numerous labs have confirmed that PGRN levels are reduced by ∼50% in FTD-*GRN* patient fibroblasts, brain, serum, and CSF (Gass et al., 2006; Ghidoni et al., 2008; Sleegers et al., 2009; Holler et al., 2016; Meeter et al., 2016), but nothing is known about the relative levels of GRNs in these patients. Using the validated GRN-detecting antibodies from Sigma (GRN-2,3) and Novus (GRN-4), we first measured mature GRN levels in primary human fibroblasts from three FTD-*GRN* patients compared with three controls (Fig. 9*A*). We found that full-length PGRN (Fig. 9*B*) as well as GRNs (Fig. 9*C*,*D*) were significantly decreased in the FTD-*GRN* fibroblasts by ∼50%, compared to controls. We next assessed GRN levels in soluble lysates of frontal cortex (Brodmann Area 9) brain tissue from five FTD-*GRN* patients compared to five age-matched controls (Table 4). To determine antibody specificity in brain, we first incubated the entire blots of soluble brain lysates with either the Sigma or Novus antibodies (Fig. 9*E*). These blots show the same prominent nonspecific bands as in the cell lysates of PGRN KO cells (Fig. 2*A*), but no specific signals for brain PGRN or GRNs at the expected MWs. Therefore, to eliminate nonspecific protein detection, we cut the membranes around 15 kDa and incubated the bottom sections with the GRN antibodies. Under these conditions, we were able to detect mature GRNs at the expected MWs which were significantly reduced by ∼60% compared to controls (Fig. 9*F-H*), similar to full-length PGRN, which was reduced by ∼40%, as measured by ELISA (Fig. 9*I*). We also observed increased levels of insoluble p62 in the FTD-*GRN* cases compared to controls, confirming that these samples were from a disease affected brain region (Fig. 9*F*). Taken together, these data demonstrate that not only PGRN, but also multiple GRNs, are haploinsufficient in FTD-*GRN* patients and raise the possibility that deficiency of GRNs in the brain contributes to the underlying pathogenic process leading to neurodegeneration.

## Discussion

In this study, we have identified antibodies that detect multiple human GRNs using a combination of expression and screening assays. Until now, antibody-based detection of endogenous human GRNs has not been reported. Using these tools, we show that mature GRNs are stably produced from intracellular PGRN and localize to LAMP1-positive lysosomes. Further, the generation of GRNs from PGRN is inhibited by SORT1 depletion, pan-lysosomal pharmacologic inhibitors, or expression of the FTD-*GRN* modifier TMEM106B. The proteolytic processing of PGRN into GRNs, mediated in part by cysteine protease activity, is conserved between humans and mice. Finally, we show that endogenous levels of multiple GRNs are haploinsufficient in FTD-*GRN* patient fibroblasts and frontal cortex, mirroring full-length PGRN.

A major contribution of this study is the development of novel tools and assays for studying human GRNs. First, we have generated constructs for each of the 7.5 human GRN proteins with and without their C-terminal linker regions (Fig. 1*A-C*; Table 2). Using these constructs, we determined the general epitopes of numerous anti-PGRN antibodies by immunoblot. We identified multiple antibodies that detect individual linker regions of PGRN as well as antibodies that recognize GRN-1, GRN-2, GRN-3, GRN-4, and GRN-5 by reducing immunoblot (Fig. 1*E*,*F*). Further, we find that the R&D AF2420 antibody recognizes all or nearly all overexpressed GRN proteins by immunocytochemistry and nonreducing immunoblot (Fig. 4*E*). One limitation to the current study is that we were only able to analyze a few GRNs in most experiments due to the limited sensitivity of some of the antibodies, which could not detect endogenous GRNs (Fig. 2*A*,*B*). Alternatively, all GRNs may not be present in cells at comparable levels. Additional higher-affinity antibodies will need to be generated to examine the endogenous expression of all GRN proteins. Second, we have developed a human *GRN* KO cell line using CRISPR-Cas9 technology (HAP1 PGRN KO) and a stable cell line that overexpresses human PGRN (HEK-PGRN) to aid in the screening and specificity of PGRN antibodies for detection of GRNs (Fig. 2). These two cell lines are crucial to verify whether antibodies recognize specific PGRN cleavage products. Overall, this suite of tools will allow for more in-depth exploration of expression, localization, and function of GRNs.

In recent years, a critical but unknown role for PGRN in proper lysosome function has emerged. FTD-*GRN* patients have half the normal levels of PGRN and abnormal accumulation of lysosomal proteins, enzymes, and storage material (Götzl et al., 2014; Ward et al., 2017), which is recapitulated in *Grn* KO mice (Ahmed et al., 2010; Kleinberger et al., 2010; Yin et al., 2010; Götzl et al., 2014; Tanaka et al., 2014). Further, a mutation in both copies of *GRN* in humans causes a lysosomal storage disease called NCL (CLN11; Smith et al., 2012; Almeida et al., 2016). Finally, PGRN is shuttled to the lysosome via SORT1 (Hu et al., 2010)- or PSAP (Zhou et al., 2015)-dependent pathways in cells and tissue. In this report, we developed a novel PGRN pulse-chase assay to study the generation, localization, and stability of GRNs (Fig. 4). Using antibodies that detect GRNs and our PGRN KO cell line, we have demonstrated that endocytosed PGRN is rapidly processed into mature, stable GRNs that localize to LAMP1-positive lysosomes (Figs. 4, 5), although we cannot rule out that GRNs may also be present in other organelles such as late endosomes. This is in line with the original description of GRNs (also known as epithelins) as heat- and acid-stable proteins that were isolated from cell and tissue lysates (Shoyab et al., 1990). Thus, we provide compelling evidence to test whether individual GRNs regulate critical lysosome functions.

The striking parallels between PGRN and PSAP strengthen our hypothesis for a functional role of GRNs in lysosomes. First, PGRN and PSAP interact with each other (Zhou et al., 2015; Nicholson et al., 2016) and with SORT1 (Lefrancois et al., 2003). PSAP, like PGRN, is processed into smaller lysosomal proteins called saposins (SAPs A, B, C, and D; Misasi et al., 2009). The individual SAPs have critical roles in lysosomal hydrolase activation needed for lipid metabolism and degradation (Schuette et al., 2001; Carvelli et al., 2015). Deficiency in PSAP or individual SAPs leads to several lysosomal storage disorders including Gaucher disease (Tamargo et al., 2012; Motta et al., 2014), Krabbe disease (Spiegel et al., 2005), and metachromatic leukodystrophy (Matsuda et al., 2001; Kuchar et al., 2009). Similarly, deficient levels of GRNs may cause lysosomal dysfunction underlying FTD or NCL neurodegeneration. Further work will be needed to determine how PGRN/GRNs and PSAP/SAPs interact and if they share any similar functions in the lysosome.

A recent study suggested that increased production of GRNs may contribute to neurodegenerative disease (Salazar et al., 2015). They report that a ∼33 kDa PGRN intermediate fragment accumulates in diseased regions of Alzheimer’s disease (AD) or FTD-TDP patient brains using an antibody generated against GRN-7 (E) (Salazar et al., 2015). This observation could imply a defect in processing of PGRN to fully mature GRNs, possibly caused by lysosome dysfunction. In contrast, we find that PGRN as well as multiple GRNs are decreased by approximately half in both FTD-*GRN* primary cells and brain tissue (Fig. 9), which is consistent with a reduction in full-length PGRN protein in FTD-*GRN* disease affected brain regions as reported previously (Chen-Plotkin et al., 2010). Therefore, it is unclear whether a loss of mature GRN proteins, an increase in intermediate GRN fragments, or both, contributes to neurodegeneration. We favor a loss-of-function hypothesis for GRNs, rather than a toxic gain-of-function, because complete loss of GRNs in humans and mice leads to NCL, characterized by severe lysosomal dysfunction and neurodegeneration (Smith et al., 2012; Almeida et al., 2016; Sargeant, 2016). Further research is necessary to clarify the role of full-length PGRN and the individual GRNs in health and disease of the nervous system.

**Figure 10.**
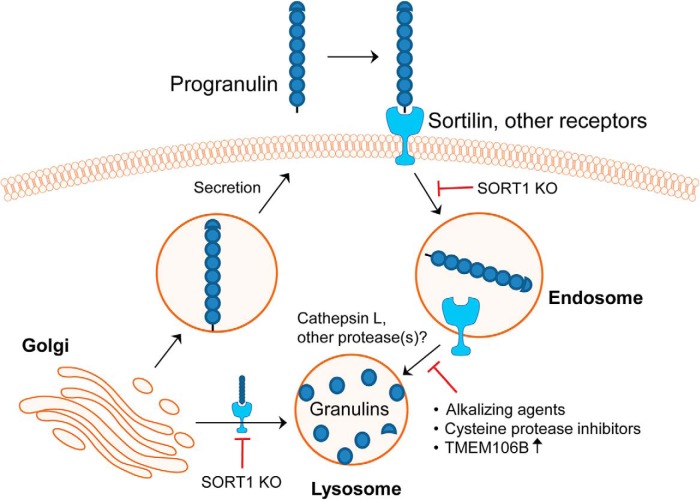
Model of intracellular processing of PGRN into stable, lysosomal GRNs. Sortilin and other receptors target endocytosed and newly synthesized PGRN to lysosomes. Within lysosomes, PGRN is proteolytically cleaved, in part, by cysteine proteases (i.e., cathepsin L) into mature, stable GRN proteins. Ablation of sortilin results in the reduced production of GRNs. Further, lysosome dysfunction caused by alkalizing agents or TMEM106B overexpression inhibits the processing of PGRN into GRNs. FTD-*GRN* patients are haploinsufficient for GRNs, which may drive lysosome dysfunction leading to neurodegeneration.

Overall, our study sheds new light on the expression, production, and localization of GRNs using newly identified and developed antibody-based tools (Fig. 10). We find that the endosomal-lysosomal pathway is a major site for the production of GRNs and is dependent on cysteine protease activity. We demonstrate that SORT1 ablation or induction of lysosome dysfunction through TMEM106B expression or pharmacologic lysosome inhibition impairs processing of PGRN into GRNs. Intriguingly, our data also suggest that PGRN is trafficked to the lysosome through additional, unknown pathways based on our observation that a) deletion of SORT1 does not eliminate production of GRNs and b) recombinant PGRN that is unable to bind SORT1 (C-TAP PGRN) is internalized by MEFs and processed into GRNs. Finally, we have found that human PGRN expressed in the mouse brain using rAAV is efficiently processed into GRNs, although we do not know the exact cell types (neurons versus glia) or site (intra or extraceullular) of GRN production in the brain. Nevertheless, the rAAV model will allow future studies to assess the localization and function of human GRNs in the mouse brain until mouse-specific GRN antibodies can be identified or developed. Because lysosome dysfunction is a hallmark of many neurodegenerative diseases, deficient production of GRNs may be a common contributor to the disease process. Indeed, reduced levels of PGRN have been linked to AD (Kelley et al., 2010; Lee et al., 2011; Minami et al., 2014; Sheng et al., 2014; Redaelli et al., 2017) and Parkinson’s disease (Chang et al., 2013; Mateo et al., 2013; Van Kampen et al., 2014; Chen et al., 2015). In the future, we will determine the functions of lysosomal GRNs and their therapeutic potential for the treatment of lysosome dysfunction and neurodegeneration.

## Note Added in Proof:

During the proof stage, it was discovered the labels for Figures 1 E and F were incorrectly listed in the text and a corrected conflict of interest statement was not included in the Early Release version, published August 9, 2017. The statement and Figure labels have now been corrected.

## Synthesis

Reviewing Editor: Julie Andersen, Buck Institute Decisions are customarily a result of the Reviewing Editor and the peer reviewers coming together and discussing their recommendations until a consensus is reached. When revisions are invited, a fact-based synthesis statement explaining their decision and outlining what is needed to prepare a revision will be listed below.

Accepted based on verbal communication with revewers
